# Macrophage migration inhibitory factor engages PI3K/Akt signalling and is a prognostic factor in metastatic melanoma

**DOI:** 10.1186/1471-2407-14-630

**Published:** 2014-08-29

**Authors:** Camila S Oliveira, Charles E de Bock, Timothy J Molloy, Elham Sadeqzadeh, Xin Yan Geng, Peter Hersey, Xu Dong Zhang, Rick F Thorne

**Affiliations:** School of Biomedical Sciences and Pharmacy, University of Newcastle, Callaghan, NSW 2308 Australia; Hunter Medical Research Institute, New Lambton Heights, Kragujevac, NSW 2305 Australia; The Kinghorn Cancer Centre and Cancer Research Program, Garvan Institute of Medical Research, Darlinghurst, NSW 2010 Australia; Kolling Institute of Medical Research, University of Sydney, St. Leonards, NSW 2065 Australia; School of Environmental and Life Sciences, Faculty of Science & IT, University of Newcastle, Ourimbah, NSW 2258 Australia

**Keywords:** Akt signalling, BRAF, Cell cycle, MIF, Melanoma, Metastasis, Prognostic factor, Proliferation

## Abstract

**Background:**

Macrophage migration inhibitory factor (MIF) is a widely expressed cytokine involved in a variety of cellular processes including cell cycle regulation and the control of proliferation. Overexpression of MIF has been reported in a number of cancer types and it has previously been shown that MIF is upregulated in melanocytic tumours with the highest expression levels occurring in malignant melanoma. However, the clinical significance of high MIF expression in melanoma has not been reported.

**Methods:**

MIF expression was depleted in human melanoma cell lines using siRNA-mediated gene knockdown and effects monitored using *in vitro* assays of proliferation, cell cycle, apoptosis, clonogenicity and Akt signalling. In silico analyses of expression microarray data were used to correlate MIF expression levels in melanoma tumours with overall patient survival using a univariate Cox regression model.

**Results:**

Knockdown of MIF significantly decreased proliferation, increased apoptosis and decreased anchorage-independent growth. Effects were associated with reduced numbers of cells entering S phase concomitant with decreased cyclin D1 and CDK4 expression, increased p27 expression and decreased Akt phosphorylation. Analysis of clinical outcome data showed that MIF expression levels in primary melanoma were not associated with outcome (HR = 1.091, p = 0.892) whereas higher levels of MIF in metastatic lesions were significantly associated with faster disease progression (HR = 2.946, p = 0.003 and HR = 4.600, p = 0.004, respectively in two independent studies).

**Conclusions:**

Our *in vitro* analyses show that MIF functions upstream of the PI3K/Akt pathway in human melanoma cell lines. Moreover, depletion of MIF inhibited melanoma proliferation, viability and clonogenic capacity. Clinically, high MIF levels in metastatic melanoma were found to be associated with faster disease recurrence. These findings support the clinical significance of MIF signalling in melanoma and provide a strong rationale for both targeting and monitoring MIF expression in clinical melanoma.

**Electronic supplementary material:**

The online version of this article (doi:10.1186/1471-2407-14-630) contains supplementary material, which is available to authorized users.

## Background

Macrophage migration inhibitory factor (MIF), so named because it inhibited the random migration of macrophages, was first discovered as a cytokine product of T lymphocytes [1, 2]. It is now known that a variety of other cells types produce MIF, including other immune cells, endocrine, endothelial and epithelial cells [3, 4]. High levels of MIF have also been reported *in vivo* in several cancer types including breast [5], lung [6] and gastric cancers [7] and the work of several groups points to a correlation between MIF expression and cancer prognosis, e.g. head and neck cancer and glioblastoma [8–10]. Moreover, findings that MIF is involved in critical pro-survival signalling pathways together with cell cycle control has provided interest in possible associations with the development and progression of cancer.

MIF protein is stored in pre-formed, cytoplasmic pools and is rapidly released in response to stimuli such as microbial products, proliferative signals and hypoxia [3, 4, 11] through a nonconventional ABC transporter pathway [12]. It is considered to be atypical of the conventional classes of cytokines with known functions extended to roles as both a hormone and an enzyme (reviewed in [3, 13]). MIF has also been shown to play a role in cell proliferation where it has been suggested to be involved in the development and progression of cancer, acting as an extracellular, pro-tumourigenic factor [14, 15].

The transmission of MIF signals occurs through a number of receptor systems, the first identified being the cell surface receptor CD74 [16]. CD74 itself lacks intracellular signalling domains [17] but it has been shown that CD44 acts as a co-receptor for CD74 to provide the means whereby MIF signals are transmitted [18]. More recently, the CXC chemokine receptors CXCR2 and CXCR4 were also identified as MIF receptors and CD74 has also been shown to form functional heteromeric receptor complexes with CXCR2 and CXCR4 [19, 20]. Depending on the cellular context, binding of MIF to its known cell surface receptors can lead to activation of two fundamental signalling axes, namely the mitogen-activated protein kinase (MAPK) pathway and PI3K/Akt signalling [14, 21–23]. In addition, the cytoplasmic pool of MIF has also been shown to exert other signalling actions.

MIF expression has also been shown to be of significance in a limited number of studies investigating melanoma biology. Higher levels of MIF mRNA were identified within isogenic variants of the human A375 melanoma selected for higher metastatic potential in nude mice [24]. Inhibition of MIF expression in the G361 human melanoma cell line resulted in inhibition of proliferation, migration and tumour-induced angiogenesis [25]. MIF production was also shown in human uveal melanoma cell lines whereby MIF prevented their lysis by NK cells [26]. Additionally, in the B16-F10 mouse melanoma model, inhibition of MIF by RNAi significantly delayed tumour establishment when injected into mice [27]. Collectively these results implicate MIF in melanoma progression, but despite this evidence, little is known on the downstream signalling pathways regulated by MIF signalling, nor has this concept been evaluated in patient studies.

In the present study, we sought to establish the primary downstream signalling pathways activated by MIF in a panel of human melanoma cell lines *in vitro* using specific knock-down studies and determine the prognostic significance of MIF expression in metastatic melanoma. Our data demonstrates that MIF is involved in melanoma proliferation and anchorage-independent growth, mediated through the activity of the PI3K/Akt pathway. We also establish that in clinical melanoma samples, MIF expression increases with metastatic progression and is correlated with survival for metastatic melanoma patients. Taken together, these results highlight the importance of the MIF-signalling axis with implications for targeted treatment approaches in melanoma.

## Methods

### Cell culture

Human melanoma cell lines were cultured in Dulbecco’s modified Eagle’s medium (DMEM; Lonza) supplemented with 5% foetal bovine serum (FBS; Sigma-Aldrich) at 37°C in a humidified atmosphere of 5% CO_2_. Me1007 and MM200 were established from primary melanomas [28], MelCV, MelRMu and MelFH were from lymph node metastases [29] and MelRM was derived from a bowel metastasis [29]. Melanoma cell lines with the prefix Mel were isolated from fresh surgical biopsies from patients attending the Sydney and Newcastle Melanoma Units. Where indicated, cell number and viability were estimated using an ADAM-MC Automatic Cell Counter (Digital Bio). The assay employs the propidium iodide (PI) method comparing suspensions of PI-stained intact cells (measuring non-viable cells) against PI-stained permeabilised cells (measuring total cells). Cell suspensions were measured in triplicate for each time point.

### Western blotting

Cells were lysed using NDE lysis Buffer (1% Nonidet P-40, 0.4% sodium deoxycholate, 66 mM EDTA, 10 mM Tris–HCl, pH 7.4) supplemented with protease and phosphatase inhibitors (Complete protease inhibitor mixture and PhosSTOP, respectively; Roche Applied Science). Protein concentrations were quantitated using BCA assay (Pierce) before electrophoresis on SDS-PAGE gels. Western blotting detection using ECL was performed as previously described [30] with bands visualized using a cooled charge-coupled device camera system (Fuji-LAS-4000, Fujifilm Life Science Systems). Primary antibodies used were: MIF (MAB289; R&D Systems); pAKT (pSer473) and total AKT (9271 and 9272, respectively; Cell Signaling Technologies); cyclin D1 and CDK4 (sc-20044 and sc-23896, respectively; Santa Cruz Biotechnology); p27 (610242; BD Transduction Laboratories™) and GAPDH (sc-25778; Santa Cruz). Secondary antibodies used were horseradish peroxidise (HRP)-conjugated anti-mouse or anti-rabbit (1706516 and 1706515 respectively; BioRad Laboratories).

### Small interfering RNA

Cells were seeded into 6-well plates at 10^5^ cells per well and allowed to reach 30-40% confluency before transfection. Synthetic siRNA duplexes were purchased from Shanghai GenePharma (PRC). Targeting sequences and validation experiments are shown in Additional file 1: Figure S1. Cells were transfected at indicated concentrations with siRNA duplexes using Lipofectamine™ RNAiMAX (Invitrogen, #13778) according to manufacturer’s instructions. Efficiency of gene knockdown was assessed by Western blotting.

### Flow cytometric analyses

DNA content analyses including quantitation of apoptotic (sub-G1) cells were performed using the propidium iodide (PI) staining method as described elsewhere [29]. The Click-iT™ EdU flow cytometry assay (Invitrogen, #C35002) was also used to determine the percentage of cells in S-phase. Briefly, three days after transfection with siRNA, cells were pulsed with 5-ethynyl-2′-deoxyuridine (EdU; 10 μM for 3 hours) before processing the cells according to manufacturer’s instructions. Receptor expression studies were performed using indirect immunostaining as previously described [31]. All flow cytometry was performed using a FACS Calibur II instrument with analyses conducted with either the Cell Quest software package v4 (Becton-Dickinson) or FlowJo v10.

### Soft agar colony formation

The ability of cells to grow under anchorage-independent conditions was measured by a soft agar colony formation assay. Briefly, 6 well plates were under-coated with 1 mL of 0.6% low melting point agar (MetaPhor®) in DMEM. Cells were harvested and 1×10^4^ cells resuspended in 1 mL of 0.3% agar/DMEM/10% FBS and the cell suspension was poured on the bottom agar layer. Plates were then incubated for 3–4 weeks before staining with 0.005% (w/v) crystal violet to visualise colonies. Bright field photomicrographs from random fields were collected using an Axiocam MRm camera fitted to Axiovert 200 inverted microscope (Zeiss) and these used to count colony frequencies. The size of colonies was estimated using the Axiovision software package (v4.8.1, Zeiss).

### *In silico*analyses

Publically available microarray gene expression data sets were sourced from the NCBI gene expression omnibus (GEO) database (http://www.ncbi.nlm.nih.gov/geo/) and normalized data used to determine the relative levels of genes of interest using methods previously described [32]. Where indicated, MIF expression was correlated with patient outcome whereby the primary end points for survival analyses were disease-specific survival (measured from the date of diagnosis to disease-specific death, or otherwise censored at the time of the last follow-up or at non-disease-related death). Time to disease-specific death was plotted using Kaplan-Meier survival curves.

## Results

### Small interfering RNA knockdown of MIF decreases melanoma cell proliferation and viability

We designed four individual siRNA oligonucleotide duplexes targeting MIF and determined the ability of each to down-regulate cellular protein levels of MIF. Western blotting analysis identified two sequences (MIF-21 and MIF-25) that were effective in reducing MIF levels in melanoma cell lines (Additional file 1: Figure S1A and B). During the course of this work, another study targeting MIF in lung cancer cells also demonstrated efficient knockdown of MIF using duplexes identical to the MIF-25 targeting sequence [33].

**Figure 1:**
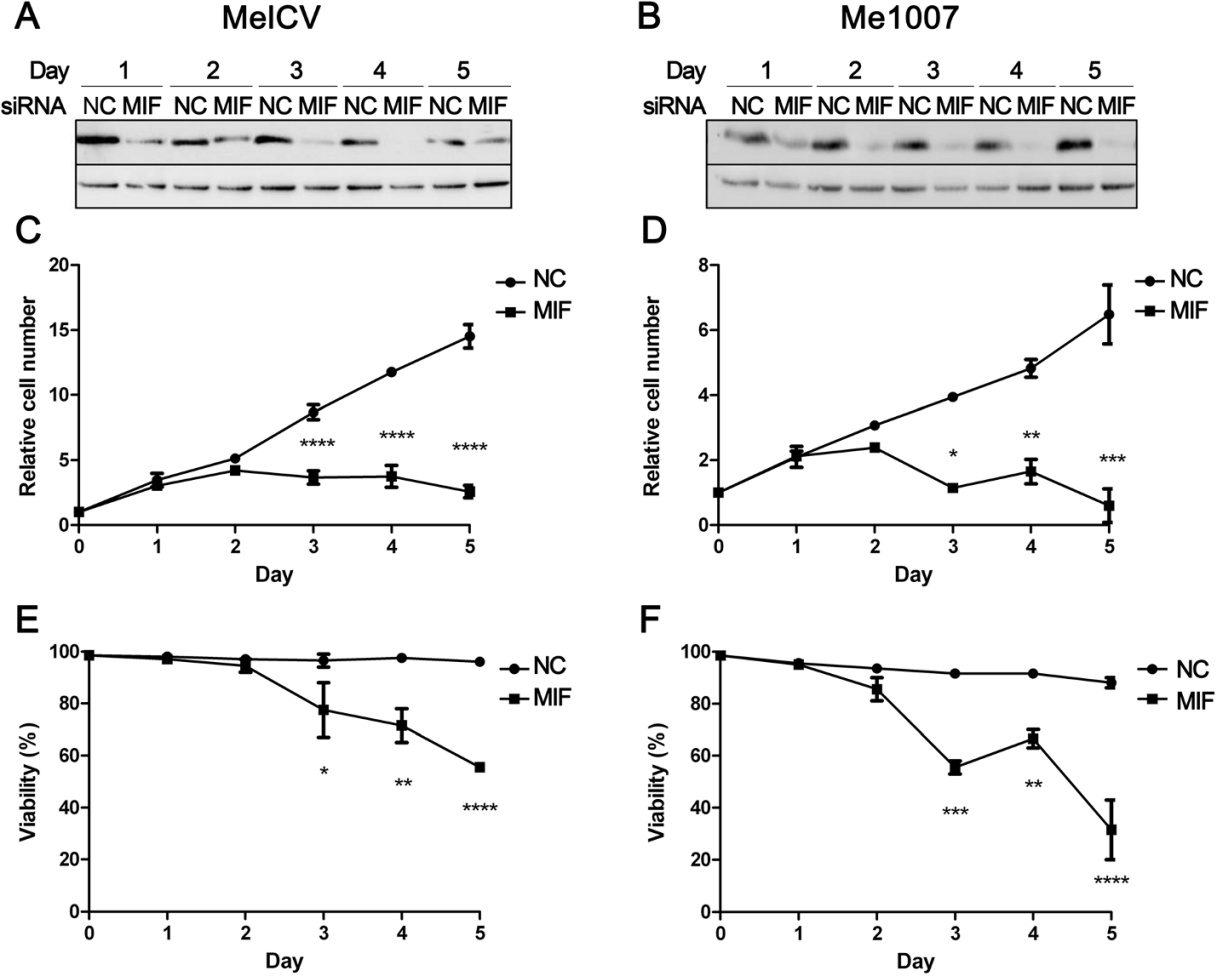
**Small interfering RNA (siRNA) knockdown of MIF decreases melanoma cell proliferation and viability.** Melanoma cell lines were transfected with MIF siRNA duplexes (50 nM MIF-25) or negative control (NC) duplexes under the same conditions. Cellular MIF levels measured in both **(A)** MelCV and **(B)** Me1007 cell lines using Western blot show sequential reductions in MIF protein after transfection that were sustained for 4–5 days. Cell number and viability were determined in corresponding samples of MelCV (**C** and **E** respectively) and Me1007 (**D** and **F**, respectively). Results show both MelCV and Me1007 showed a significant reduction in the cell number starting from day 3 after transfection with viability also reduced in a time-dependent manner. Values are mean +/-SEM (n = 3, ****p < 0.0001; ***p < 0.001; **p < 0.01; *p < 0.05 comparing between NC and MIF siRNA transfected cells using Student’s *t*-test).

To determine the effects of MIF knockdown on melanoma cell growth, cell number and viability were measured each day over a 5 day period. Comparison of MelCV and Me1007 melanoma cells transfected with MIF-25 siRNAs confirmed a substantial reduction in the total MIF protein measured in cell lysates relative to negative control (NC) siRNA treatment (Figure 1A and B, respectively). For both cell lines, the total number of cells began to decrease after 3 days of MIF knockdown (Figure 1C and D) and this was accompanied by significant reductions in cell viability (Figure 1E and F). Both active siRNA duplexes (MIF-21 and MIF-25) promoted equivalent biological responses (Additional file 1: Figure S1C and data not shown) indicating that depletion of endogenous MIF can significantly compromise the proliferative capacity and viability of melanoma cells in culture.

### MIF depletion retards melanoma cell cycle progression and prevents anchorage-independent growth

**Figure 2:**
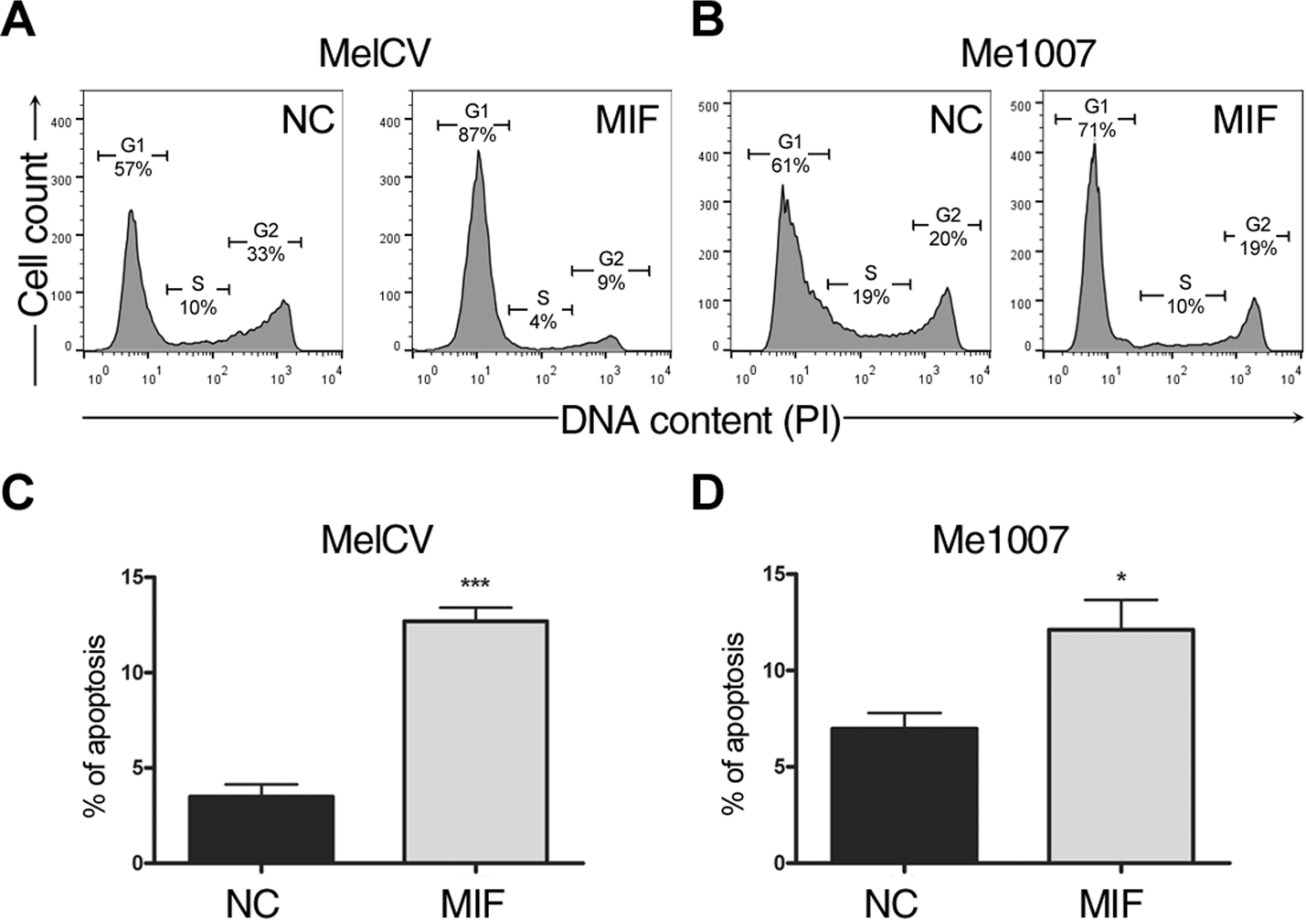
**Effects of MIF knockdown on cell cycle and apoptosis.** DNA content analysis using flow cytometry was performed on **(A)** MelCV and **(B)** Me1007 cells after 3d of transfection with control (NC) or MIF siRNA duplexes. Representative profiles show the estimated percentages of cells in gates representing G0/1 (G1), S and G2/M (G2) phases. Only cells with intact DNA contents (2n-4n) were analysed. Apoptotic rates were also estimated as the percentage of total cells present in the sub-G0/G1 region. The percentage of apoptotic cells was estimated from **(C)** MelCV and **(D)** Me1007 cells after 3d of transfection with MIF or control siRNA duplexes. The histograms represent the means +/-S.E.M. from 3 replicates (***p < 0.001 and *p < 0.05 comparing between treatments using Student’s *t*-test). Similar results were obtained in three independent experiments.

To address the mechanism whereby MIF depletion was associated with reduced cell growth and viability, DNA contents were measured by flow cytometry. Representative profiles of MelCV and Me1007 cells are shown after treatment with control NC siRNA or siRNA against MIF (Figure 2A and B). As shown, MIF depletion resulted in an increased number of cells in the G0/1-phase in both cell lines (i.e. from 57% to 87% and 61% to 71% for MelCV and Me1007 cells, respectively). For MelCV cells there was a reduction in the number of cells recorded in the S- and G2/M phases after MIF depletion (Figure 2A) while in Me1007 cells the major effect appeared to be a reduction in the percentage of cells in S-phase (Figure 2B).

In addition to the changes in cell cycle parameters, we also assessed whether cells were undergoing increased rates of apoptosis as suggested by the decreased viability observed in Figure 1. An estimate of apoptosis was determined from the DNA content analysis as the number of cells appearing in the sub-G0/1 region, i.e. cells with DNA content of less than 2n. This analysis showed that the basal level of apoptosis in control cultures increased ~2-3 fold when the MelCV or Me1007 were treated with siRNA against MIF for 3 days (Figure 2C and D). Taken together with the results of Figure 1, these data suggest that reduced cell growth occurred as a result of cells accumulating in the G0/1 phase and that the progressive decline in cell viability was caused in part by increased rates of apoptosis.

**Figure 3:**
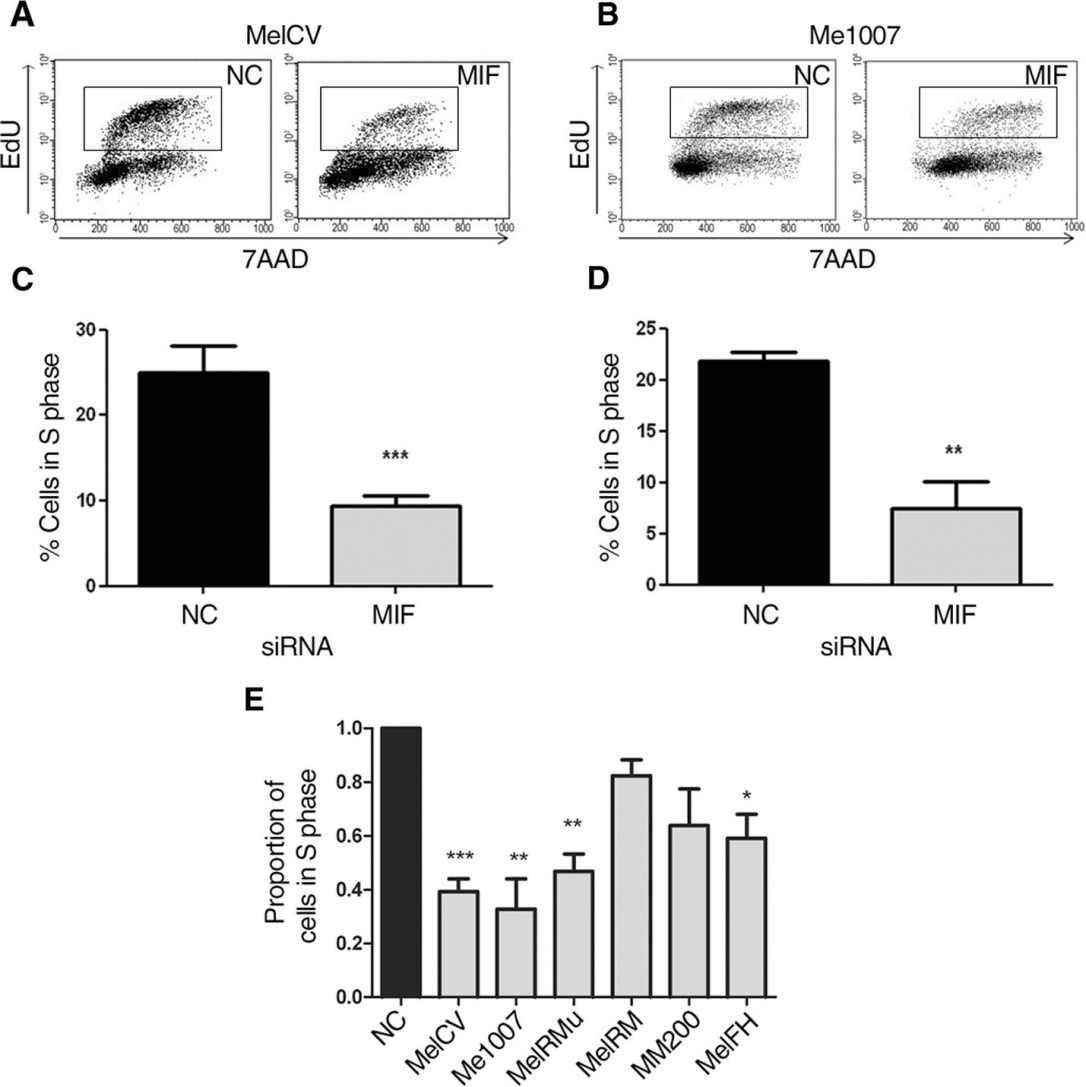
**Effects of MIF knockdown on the S-phase of the cell cycle.** The Click-iT™ EdU flow cytometry assay was used to measure the numbers of cells entering S-phase over a 3 h period. Dual parameter plots compare cellular DNA content (7AAD) against Edu incorporation with S-phase cells denoted by the inset box in each plot. Representative analyses for **(A)** MelCV and **(B)** Me1007 cells are presented after 3d of transfection with control (NC) or MIF siRNA duplexes. Quantitation of the percentage of cells in S phase in **(C)** MelCV and **(D)** Me1007 cell lines after MIF knockdown or treatment with control siRNA duplexes. **(E)** Analyses in **(C)** and **(D)** were repeated for an additional 4 melanoma cell lines. Values shown represent the proportion of cells entering S phase after MIF siRNA treatment normalised against NC siRNA treatment. MIF depletion significantly reduced the number of cells entering S-phase for 4/6 cell lines (means +/-S.E.M. from 5 independent experiments, ***p < 0.001 **p < 0.01 *p < 0.05 comparing between treatments using Student’s *t*-test).

To better define the effects of MIF knock down in melanoma cell lines, particularly their decreased proliferative capacity, the ability of cells to enter the S-phase of the cell cycle was measured using the Click-iT™ EdU flow cytometry assay. Click-iT analysis of MelCV and Me1007 cells treated with MIF siRNA showed a clear reduction in cells entering S-phase (Figure 3A and B). The results from 5 independent experiments show that inhibition of MIF expression significantly reduces the percentage of cells in S phase compared to negative control siRNA transfection for both the MelCV and Me1007 melanoma cell lines (Figure 3C and D, respectively). MIF depletion also significantly reduced the number of cells entering S-phase in four of six melanoma cell lines examined (Figure 3E) suggesting the proliferative capacity of the majority of melanomas have some degree of reliance on MIF expression.

**Figure 4:**
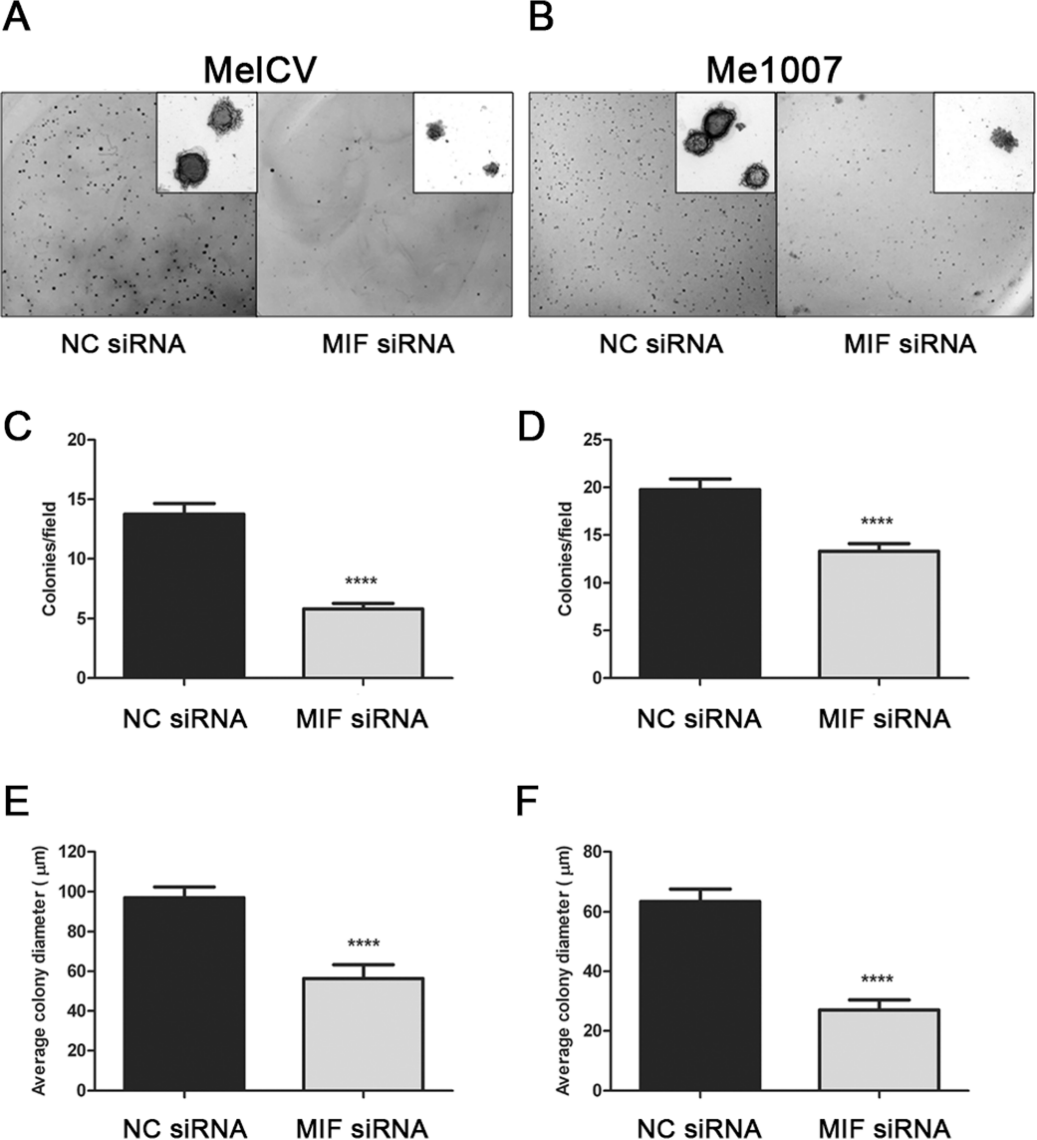
**MIF knockdown decreases anchorage-independent colony formation.** Three days after transfection using siRNA duplexes, melanoma cells were harvested and seeded into soft agar and permitted to form colonies according to the Methods. Representative low power photomicrographs demonstrating the relative abundance of colonies formed by **(A)** MelCV and **(B)** Me1007 cells after control NC or MIF siRNA knockdown (the insets show colonies in detail under higher magnification). The frequency and size of colonies were then estimated for MelCV **(C) (E)**, respectively and Me1007 **(D) (F)**, respectively where there was a significant reduction in colony numbers and size after MIF knockdown. Values are mean +/-S.E.M. (n = 3, ****p < 0.0001 compared to siNC transfected cells using Student’s *t*-test).

In addition to altered cell proliferation, the ability of cells to undergo anchorage-independent cell growth is another hallmark of cancer. Loss of MIF expression in MelCV and Me1007 melanoma cell lines resulted in significantly less colonies in both cell lines compared to controls (Figure 4A–D). Moreover, the colonies formed after MIF knockdown were also significantly smaller than controls (Figure 4E and F). Taken together, these results provide further evidence that MIF expression regulates both cell cycle entry and the clonogeneic capacity of melanoma cells *in vitro*.

As part of our studies we also sought to determine whether the individual responses of cell lines to MIF could be explained by expression of the known cellular receptors for MIF that comprise CD74 and its co-receptor CD44, along with the chemokine receptors CXCR2 and CXCR4. Analysis by both Western blotting and flow cytometry showed that all six melanoma lines expressed both CD44 and CXCR4 while none expressed CXCR2 (Additional file 1: Figures S2 and S3). All lines varied in expression of CD74 but there was no clear correlation between expression levels and sensitivity of individual cell lines to MIF depletion. Interestingly, there was also no correlation between the V600E BRAF status of each line and sensitivity to MIF depletion since the V600E BRAF positive cell lines MelCV and MelRMu were amongst the most sensitive to MIF depletion (refer Discussion).

### MIF regulates PI3K/Akt signalling and key cell cycle proteins in melanoma cell lines

**Figure 5:**
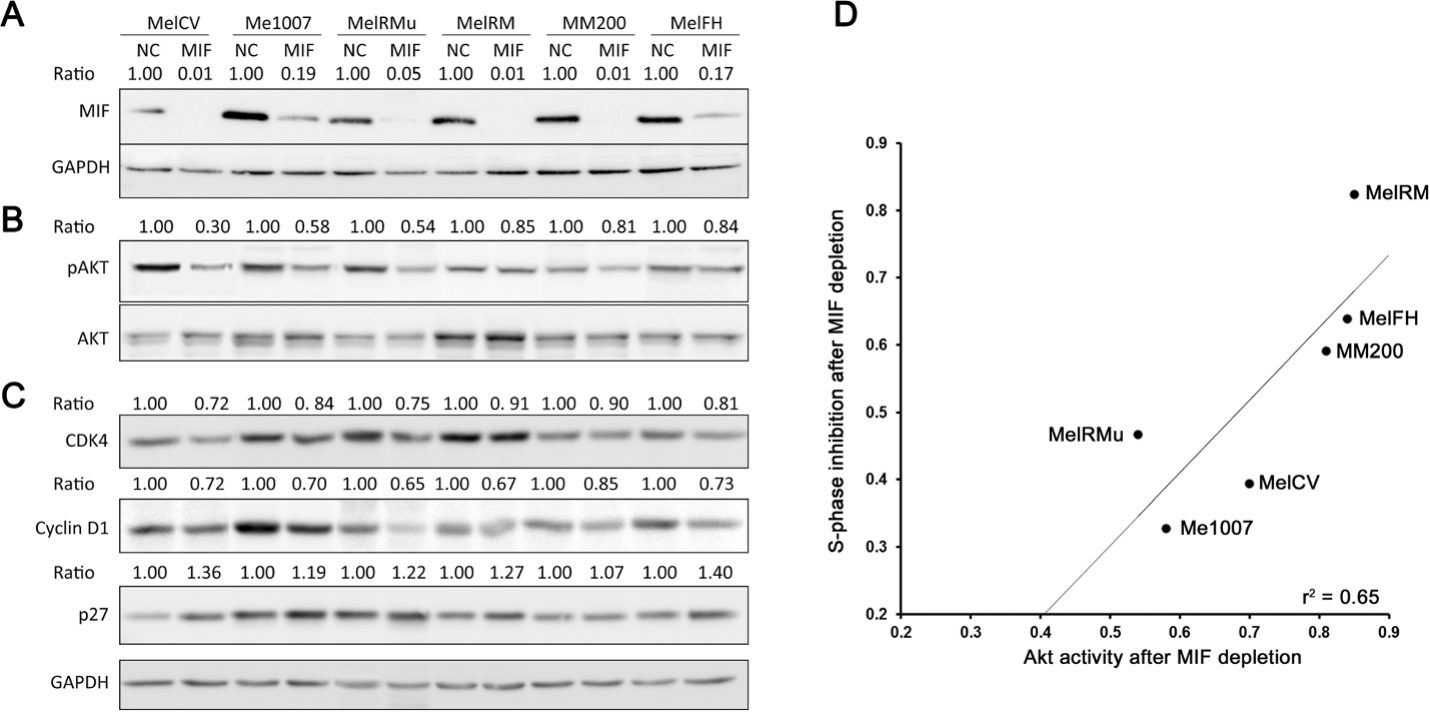
**MIF modulates the PI3K/Akt signalling pathway in melanoma cell lines.** MIF expression was depleted in a panel of six melanoma cell lines as described before. Three days after transfection, the cellular levels of MIF, Akt and key cell cycle regulators were measured using Western blotting. Densitometric signals were calculated for each protein band using the MultiGauge software package (Fuji Life Sciences) and used to determine relative expression following depletion of MIF. **(A)** Representative Western blots showing specific immunoreactive bands for MIF in the indicated melanoma cell lines. The normalised ratio of expression of MIF was determined by dividing MIF levels after depletion against control levels. **(B)** Levels of phosphorylated Akt (Ser 473) and total Akt where the ratio represents the relative phospho-Akt levels compared to total Akt. **(C)** Levels of the cell cycle regulators cyclin D1, CDK4 and the cyclin-dependent kinase inhibitor p27. Each ratio was determined by dividing the optical density of the specific band by the GAPDH value. The results shown were consistent across at least 3 independent experiments. **(D)** Dual parameter plot comparing the degree of inhibition of proliferation after MIF knockdown (effects on the proportion of cells entering S-phase; Figure 3) with the corresponding effects on the level of Akt activity observed (relative levels of pAkt; Figure 4A).

Having established that MIF exerts effects on the cell cycle entry and the clonogenic capacity of melanoma cells, we sought to determine which pathways are activated downstream of MIF. Signalling through the PI3K/Akt pathway is well established to play an important role in melanoma progression [34–36] but to date no link has been made between MIF expression and regulation of the PI3K/Akt pathway in this setting. Therefore, we examined the effects of MIF knockdown on the expression of key Akt-signalling components in melanoma cells. With respect to the proliferative capacity of melanoma cells, it was observed from Figure 3 that four melanoma cell lines tested were sensitive to MIF depletion (MelCV, Me1007, MelRMu and MelFH) and another two (MelRM and MM200) were comparatively resistant. All six melanoma cell lines were subjected to treatment with MIF siRNA with knockdown of MIF protein after three days of transfection confirmed relative to controls using Western blotting (Figure 5A). Analysis of Akt phosphorylation status in cell lysates indicated a strong reduction in MelCV, Me1007 and MelRMu cells (~40-70% of controls) as a consequence of MIF knockdown with a lesser reduction observed in MelFH, MM200 and MelRM cell lines (~20% of controls; Figure 5A).

We then sought to determine whether there was a direct correlation between the relative effects of MIF knockdown on cell proliferation (inhibition of S phase; Figure 3E) and the relative levels of Akt activation for each cell line. There was a demonstrable positive correlation where cell lines most sensitive to MIF depletion also had the greatest change in Akt activity and vice versa (Figure 5B). Further analysis of the downstream cell cycle modulators known to be influenced by Akt signalling was also undertaken. CDK4 and cyclin D1 involved in G1/S transition also showed some level of inhibition across the six cell lines, whereas the expression of cyclin-dependent kinase inhibitor, p27, was relatively increased in most of the cell lines following MIF depletion. On balance these results support the notion that Akt-signalling is down-regulated in response to MIF knockdown with the degree of sensitivity to MIF depletion commensurate with the inhibitory effects observed on the Akt pathway.

### Expression levels of MIF in melanoma metastases are associated with disease progression

**Figure 6:**
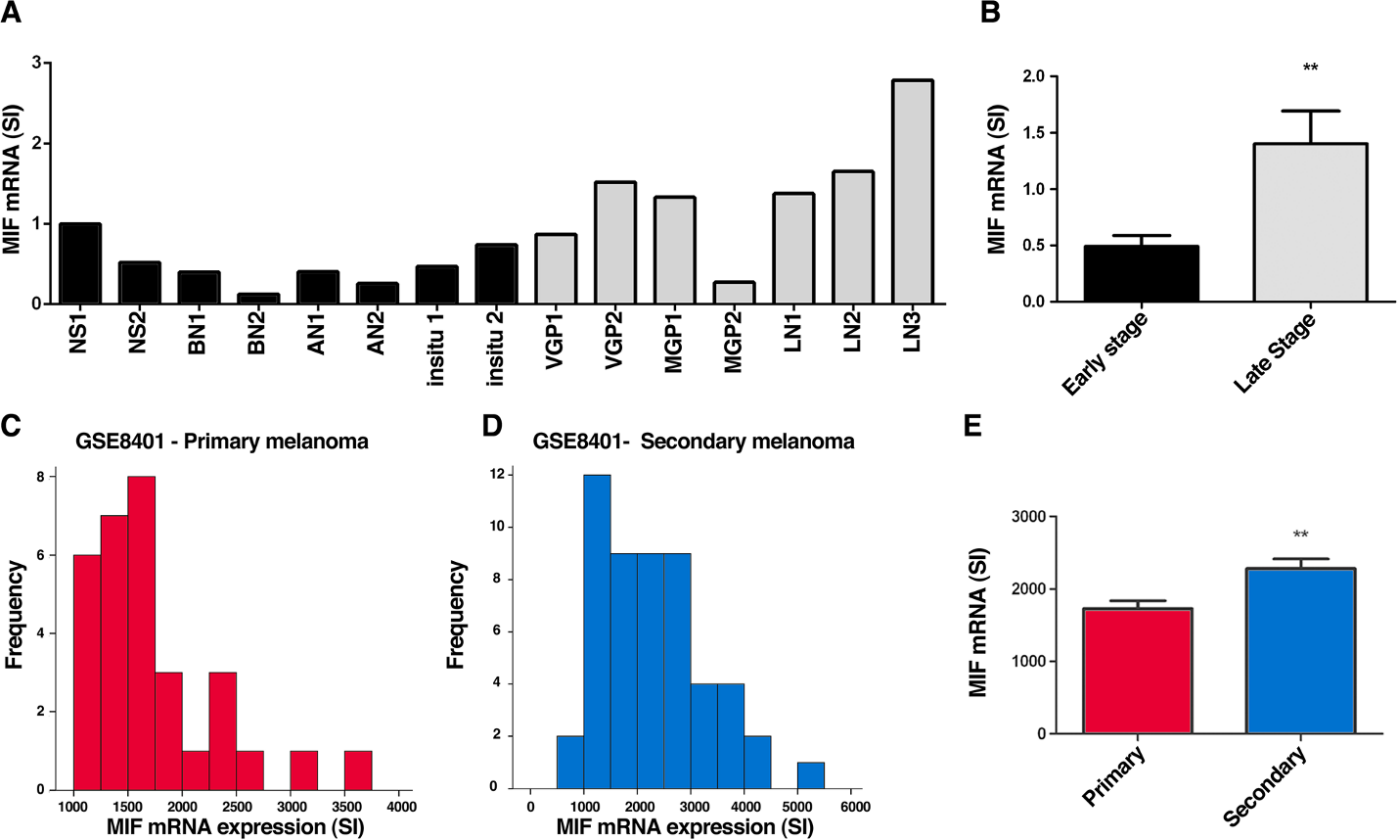
**MIF expression increases during progression of melanocytic lesions to advanced stage melanoma. (A)** Levels of MIF mRNA expression are compared in two normal skin tissue samples (NS1; NS2), benign naevi (BN1; BN2), atypical naevi (AN1; AN2), melanomas *in situ* (in situ1; in situ2), VGP melanomas (VGP1; VGP2), MGP melanomas (MGP1; MGP2), and the three MGP melanoma-positive lymph nodes (LN1; LN2; LN3). Data represent normalised levels extracted from GEO dataset GSE4587. **(B)** Average MIF expression levels were higher in “advanced stage” samples compared to the “early stage” samples (mean +/-S.E.M. of early stage (n = 8) and advanced stage (n = 7) from **(A)**, **p < 0.01 using the Mann–Whitney test). **(C/D)** Frequency distribution histograms of MIF transcript expression in tissues of primary melanoma or metastatic melanoma. Analyses were conducted on expression microarray data (GEO dataset GSE8401) from melanoma tissues of patients with progressive disease collected as 31 cases of primary melanoma **(C)** and 52 cases of metastatic melanoma **(D)**. **(E)** Analysis of MIF expression data from **(C)** and **(D)** shows the levels are higher in metastatic melanoma compared to primary melanoma samples. (Values are mean +/-S.E.M. from primary and metastatic melanoma cases respectively, **p < 0.01 using the Mann–Whitney test).

After establishing that MIF expression is important for the maintenance of melanoma cells *in vitro,* we investigated whether MIF expression levels were also elevated and/or associated with clinical outcomes in melanoma. Firstly, we independently performed *in silico* analyses of expression microarray data comparing the relative transcript levels of MIF in staged melanoma against normal skin and naevi (samples of normal skin, benign naevi, atypical naevi, melanoma *in situ*, vertical growth phase (VGP) and metastatic growth phase (MGP) melanoma, melanoma-positive lymph nodes (LN); deposited as GEO dataset GSE4587 [37]). The expression levels of MIF were determined as normalized intensity values (GeneSpring 7.1, Silicon Genetics) for each sample (Figure 6A). The level and pattern of MIF expression show a general increase in MIF levels associated with disease progression. Dividing the samples into two groups, “early stage” (normal skin, benign and atypical naevi, melanoma *in situ*) and “advanced stage” (VGP and MGP melanomas, LN) demonstrated a statistically significant increase in MIF expression in “advanced-stage” tissue samples compared to the “early-stage” group (Figure 6B). To substantiate this finding, further analyses were conducted on the data set generated by Xu *et al.* [38] consisting of eighty-three fresh biopsies from melanoma patients (profiled using the Affymetrix U133A microarray platform; GEO accession number GSE8401). The distribution of MIF expression for primary melanoma (n = 31) and metastatic melanoma (n = 52) are shown in Figure 6C and D, respectively, where MIF mRNA levels appear relatively increased in metastatic samples. Analysis of average levels in each group showed a statistically higher level of MIF in metastatic melanoma compared to primary tumour samples (Figure 6E). Collectively these findings support the notion that MIF expression is up-regulated during melanoma progression.

**Figure 7:**
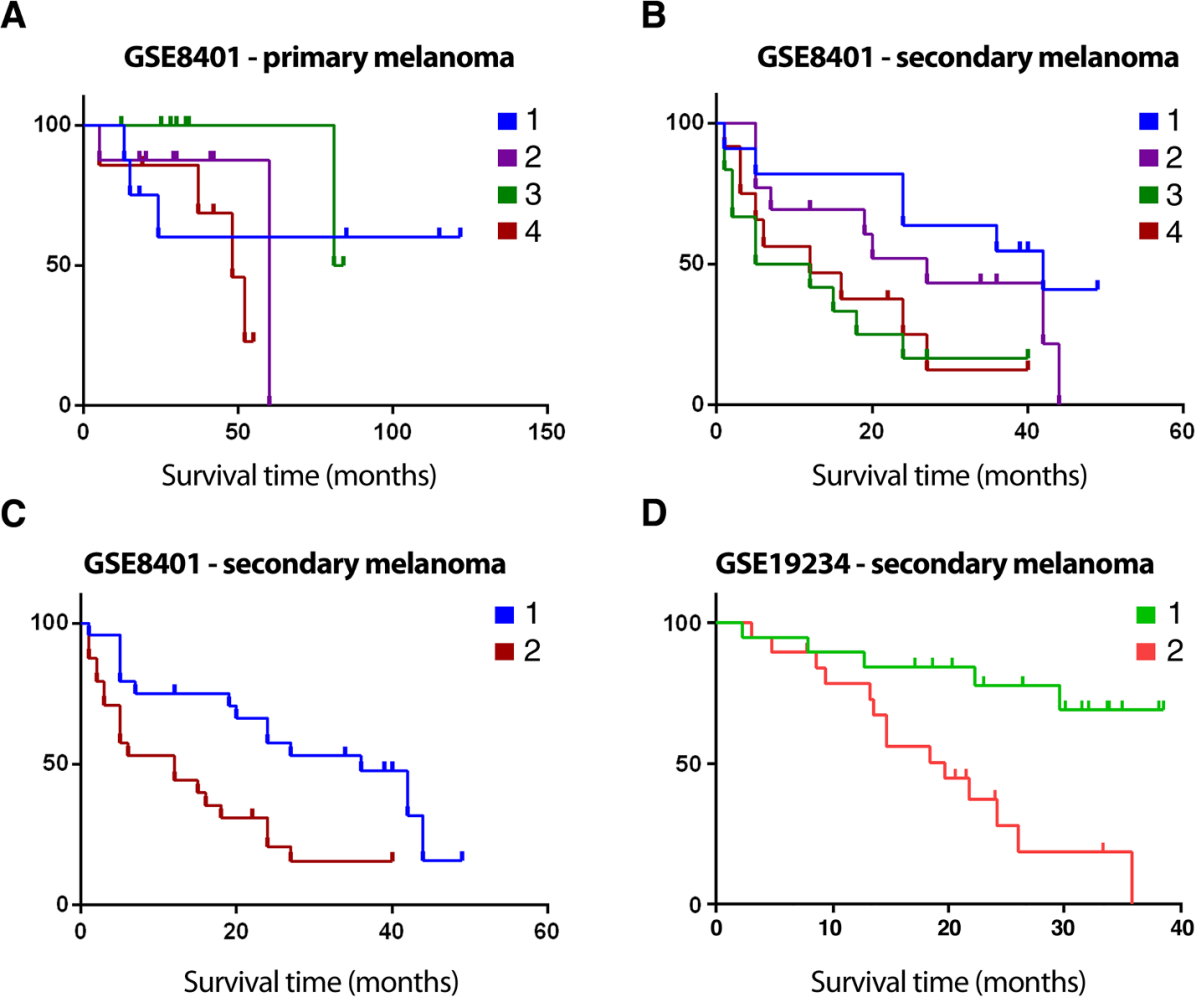
**High MIF expression in metastatic melanoma lesions is associated with worse outcomes.** Kaplan-Meier survival curves generated on the basis of quartiles of MIF tumour expression levels in **(A)** primary and **(B)** metastatic melanoma tissues correlated against disease-specific survival (GSE8401 dataset). **(C)** Kaplan-Meier survival curves generated for low and high expressing samples of metastatic melanoma tissues using a 50% cutoff level (GSE8401 dataset). **(D)** Analysis conducted as per **(C)** on an independent dataset of metastatic melanoma tissues (GSE19234, n = 38 cases).

We then sought to establish whether levels of MIF expression in melanoma were predictive of outcome by exploiting expression microarray data associated with clinical outcomes. Since the levels of MIF differed between primary and metastatic melanoma, analyses were conducted on each classification where tumour biopsies were initially segregated into quartiles of MIF expression ranging from low to high values. Kaplan-Meir plots of MIF levels against survival appeared to show no predictive significance of MIF levels in primary melanoma tumours (Figure 7A) whereas a clear trend was evident for metastatic samples where the first and second quartiles segregated from the third and fourth quartiles (Figure 7B). Further analysis of the data using a 50% cut-off showed that high levels of MIF in metastatic disease conferred significantly poorer outcome compared to those tumours expressing lower levels of MIF mRNA (univariate Cox regression; hazard ratio = 2.946; 95% confidence interval 1.440-6.029; p = 0.003; Figure 7C). The same analyses conducted on the primary melanoma data showed no significant relationship between MIF expression in and survival (hazard ratio = 1.091; 95% confidence interval 0.312-3.809; p = 0.892). As further validation, the same analysis of an independent dataset of metastatic melanoma tissues (GSE19234; [39]) also indicated significantly worse outcomes for patients whose tumours expressed higher levels of MIF (univariate Cox regression; hazard ratio = 4.600; 95% confidence interval 1.6-12.9; p = 0.004; Figure 7D). Thus in patients where metastasis had already occurred, those cases with tumours displaying the highest levels of MIF progressed faster.

## Discussion

To date, apart from two studies each using a single cell line [24, 25] the role of MIF in the context of human cutaneous melanoma has not been intensively studied. In the present report we adopted an siRNA-based strategy to examine the function of endogenous MIF expression in multiple human melanoma cell lines. In MelCV and Me1007 cell lines, MIF knockdown resulted in significantly reduced cell number and viability over 6 days, indicating that endogenous MIF expression could be generally required for the growth of melanoma cells. The reduced cell numbers corresponded to the increased accumulation of cells in G0/1 and a decrease of cells in S-phase. Moreover, accounting for the successive reduction in the number of viable cells during the experiment, there was an increased proportion of apoptotic cells following MIF depletion. Similar findings were also obtained when considering anchorage-independent growth where it was shown that MIF siRNA transfection significantly compromised the number and size of colonies formed by melanoma cells.

To better understand the role of MIF expression in melanoma cells, further quantitative assays were employed on six different melanoma cell lines. Cell proliferation after MIF knockdown was further explored using the Click-iT assay, a sensitive and quantitative assay which measures the cell cycle. In particular the assay provides an accurate measure of the number of cells entering S-phase in a fixed time period. This analysis showed that MIF knockdown significantly reduced cells transitioning to the S-phase in four of the six melanoma cell lines suggesting the proliferative capacity of the majority of the melanoma cell lines studied have some degree of reliance on MIF expression. In agreement with these findings, work from several authors have shown that MIF is involved in cell cycle regulation in different cancer cells [14, 23, 40], and MIF knockdown can cause G1 arrest by inhibiting G1/S transition [41]. At least for the MelCV and Me1007 lines examined in detail, MIF depletion was clearly cytostatic but also compromised cell viability. Collectively this reinforces the idea that MIF signalling displays potential as a pathway that could be targeted for melanoma treatment.

Leading on from these findings the question is raised as to how MIF functions in this setting. We could not establish that the sensitivity of individual melanoma cell lines to MIF depletion resulted from the differential expression of known MIF receptors (i.e. CD74/CD44 and/or CXCR4). We also considered the responsiveness of cells lines in the context of known downstream signalling pathways. It is well established that MIF function is associated with two major pro-survival pathways, namely the MAPK and PI3K/Akt signalling pathways, each known to be important in melanoma. Indeed, oncogenic MAPK signalling through ERK (RAS-RAF-MEK-ERK) is constitutively activated in the majority of melanomas [42, 43] with aberrant activation frequently stemming from activating mutations in BRAF. Of these, the most common BRAF mutation occurring in ~50% of all melanomas comprises a glutamic acid to valine substitution at position 600 (V600E) [43–45]. Pharmacological inhibition of the RAF/MEK/ERK pathway, in particular, via inhibition of mutated and activated BRAF, has therefore appeared as a promising strategy for treatment. This has led to the development of mutant-BRAF-specific inhibitors [46–49] that have shown promising results in clinical trials [47, 50, 51]. In our study, of the melanoma cell lines tested for the effects of MIF inhibition, three express wildtype BRAF (Me1007, MelRM and MelFH) while the others bear the BRAF V600E mutation (MelCV, MelRMu and MM200 [52]). Two of the three lines most sensitive to MIF depletion are BRAF mutants (MelCV and MelRMu) indicating the effects of MIF signalling in melanoma were likely outside this pathway. This observation has important therapeutic implications in patients that are resistant to mutant BRAF inhibitors (e.g. [53]) whereby MIF depletion/targeting could be used as an alternative strategy.

Alongside the MAPK pathway, constitutive activation of the PI3K/Akt pathway is also important in a high proportion of melanoma cases [54–56]. Such activation is frequently associated with down-regulation or loss of PTEN phosphatase that antagonises PI3K signalling. Absent or decreased PTEN expression occurs in up to 90% of primary melanomas with mutations of PTEN or loss of heterozygosity at the PTEN locus accounting for this deficiency [57, 58]. Transcriptional repression of the gene by promoter methylation also occurs in a high proportion of metastatic melanomas [59, 60]. In addition to PTEN, other major elements of the PI3K/Akt pathway are found to be amplified or mutated in melanoma. Akt3 expression is increased as a result of elevated gene copy number in ~50% and ~70% of primary melanomas and metastatic melanomas, respectively [36]. PI3K/Akt pathway activation in melanoma can also occur through mutational activation of PI3KCA along with the mutational activation of upstream receptor tyrosine kinases such as c-KIT and EGFR [61, 62]. Given the previous links established between MIF and Akt signalling as described in the Introduction, we focussed our efforts on investigating Akt as the likely major pathway downstream of MIF in melanoma.

Akt activation can stimulate proliferation through multiple downstream targets that affect cell-cycle regulation [63]. For example, Akt can phosphorylate p27 (Kip1) cyclin-dependent kinase inhibitor, preventing its localisation to the nucleus and attenuating its cell-cycle inhibitory effects [64–66]. In addition, Akt also serves to phosphorylate and inactivate GSK3. As GSK3 phosphorylates cyclin D and cyclin E and targets them for proteasomal degradation [67, 68], inhibition of GSK3 by Akt thereby acts to stabilise key cyclins involved in cell cycle entry. In the current study, MIF knockdown resulted in decreased Akt phosphorylation in all melanoma cell lines tested, albeit to varying degrees. This effect was accompanied by a reduction in expression of cyclin D1 and cyclin dependent kinase 4 (CDK4), and an increased expression of p27. Moreover there was a correlation between the effects of MIF knockdown and the degree of Akt activation amongst individual cell lines. Collectively this suggests that activation of the Akt pathway is one of the major mechanisms whereby MIF contributes to cell cycle regulation in melanoma. Since the overall importance of the PI3K/Akt pathway to melanoma biology cannot be understated, these findings imply clinical significance of MIF signalling in this disease.

Supporting this notion, a previous study of melanocytic tumours showed MIF mRNA and protein levels were high in malignant tumours while expression was significantly lower in benign naevi [69]. Here we verified these data using independent expression microarray datasets where collectively these findings support the general concept that MIF is differentially expressed between non-malignant and malignant lesions with increased expression during melanoma progression. Despite these observations and previous work associating increased MIF with enhanced melanoma growth and metastasis in nude mice [24], the clinical significance of MIF tumour levels has surprisingly not been previously examined. Our *in silico* analyses of GEO datasets now reveal significant correlations between MIF expression and patient outcome. While MIF levels in primary tumours had no bearing on patient outcomes there was a clear indication that high MIF expression levels in metastatic lesions were significantly associated with shorter survival times. Indeed, in the GSE19234 cohort, ~70% of patients whose tumours has lower MIF expression remained alive approaching 40 months of clinical follow-up.

One caveat to consider when linking our *in vitro* findings to the clinical setting is the inherent complexity of tumour tissues *in vivo*. It seems probable that MIF expression in melanoma cells has an impact upon their proliferative capacity *in vivo* but whether the MIF gene expression detected in clinical samples is wholly tumour derived is not entirely clear. Tumour tissue comprises a non-homogenous mixture of tumour and stromal cells including variable amounts of infiltrating immune cells. In breast cancer, MIF is expressed in both tumour cells and stromal cells, including tumour-associated macrophages [5]. Indeed, MIF is a key cytokine in both innate and adaptive immune cells [3, 4] and thus infiltrating immune cells must also be considered as an intra-tumoural source of MIF. Some breast cancer cells respond to exogenous MIF by triggering a massive burst of MIF secretion suggesting auto- or paracrine regulation of MIF [70]. Finally, it is already well established that interactions between the tumour and its microenvironment play an important role in influencing the behaviour of tumour cells. Again in breast cancer, it was found that MIF was highly upregulated in tumours cells when they were co-cultured with macrophages. In turn, increased MIF secretion by tumour cells contributed to metalloproteinase production by the macrophages and this augmented the invasive potential of the tumour cells [70]. Similarly it is known that tumour-associated macrophages can enhance melanoma growth though secreted factors (e.g. [71]) and equally there are other infiltrating cells such as lymphocytes [72] which are also potential sources of MIF. However the relative importance of MIF production in melanoma tumour cells versus stromal cells remains to be established.

## Conclusions

Our results establish the concept that high MIF expression levels in metastatic melanoma is associated with faster relapse and death. Through *in vitro* analyses, a mechanism in suggested where MIF expression is associated with activation of Akt signalling and promotion of melanoma proliferation and survival. In the current environment where mutant BRAF status dominates the clinical approach, the effects of MIF signalling are notionally independent of BRAF mutational status. Thus, in addition to the potential of MIF as a prognostic marker, these data highlight the potential utility for MIF and its signalling axis as a treatment target in this disease.

## Electronic supplementary material

Additional file 1: Figure S1: Efficacy of siRNA duplexes targeting MIF in the inhibition of cell growth. **Figure S2.** Expression of MIF and its receptors CD44 and CD74 in a human melanoma cell line panel. **Figure S3.** Chemokine receptor expression in human melanoma cell lines. (PDF 542 KB)

## Abbreviations

MIF: Macrophage inhibitory factor.
