# Supplementary material for: Macrophage migration inhibitory factor engages PI3K/Akt signalling and is a prognostic factor in metastatic melanoma
Source: BMC Cancer. 2014 Aug 29;14:630. doi: 10.1186/1471-2407-14-630 (PMC4155090; doi:10.1186/1471-2407-14-630)
Supplement: Supplementary file 1 — Additional file 1: Figure S1: Efficacy of siRNA duplexes targeting MIF in the inhibition of cell growth. Figure S2. Expression of MIF and its receptors CD44 and CD74 in a human melanoma cell line panel. Figure S3. Chemokine receptor expression in human melanoma cell lines. (PDF 542 KB) [file 12885_2014_4806_MOESM1_ESM.pdf]

A

Designation	Duplex sequences	*Targeting region
MIF 21	5’-UGGUGUUUACGAUGAACAUTT-3’	104-122
	5’-AUGUUCAUCGUAAACACCATT-3’	
MIF 25	5’-UUGGUGUUUACGAUGAACAUCGGCA–3’	99-123
	5’-UGCCGAUGUUCAUCGUAAACACCAA-3’	
MIF 36	5’-AUAGUUGAUGUAGACCCUGUCCGGG-3’	370-394
	5’-CCCGGACAGGGUCUACAUCAACUAU-3’	
MIF 37	5’-UUCCAGCCCACAUUGGCCGCGUUCA-3’	402-426
	5’-UGAACGCGGCCAAUGUGGGCUGGAA-3’	
NC	5’–UUCUCCGAACGUGUCACGUTT–3’	N/A
	5’-ACGUGACACGUUCGGAGAATT-3’	

* against MIF (NCBI Reference Sequence: NM_002415.1)

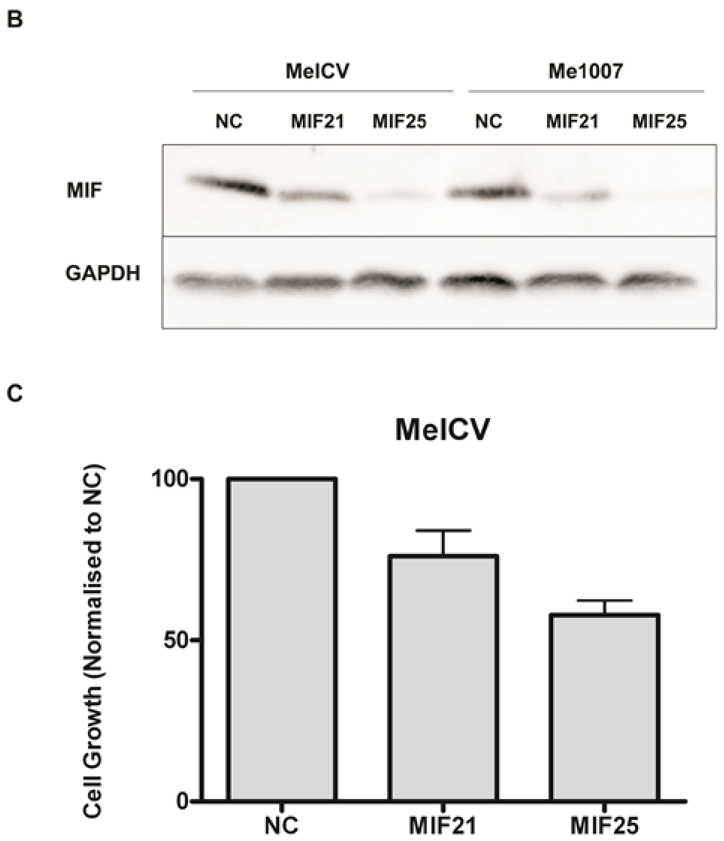

Figure S1. Efficacy of siRNA duplexes targeting MIF in the inhibition of cell growth. (A) Sequences of four siRNA duplexes designed to target MIF along with the negative control (NC) duplexes used. (B) Western blot analysis of MIF in total cellular lysates after treatment with the indicated siRNA duplexes from (A). MelCV and Me1007 cells were transfected according to the Methods and cell lysates collected after 3 days. Western blotting against GAPDH confirmed equal protein loading of each sample. The results of two independent transfections are shown indicating both MIF-21 and MIF-25 could deplete MIF but the effectiveness of MIF-25 was far more substantial. (C) Cell growth assays were conducted using the MTS assay under the conditions described for (B). Data were normalised to growth of NC-treated cells. The results represent the average of two independent transfections each analysed in triplicate +/- SDs.

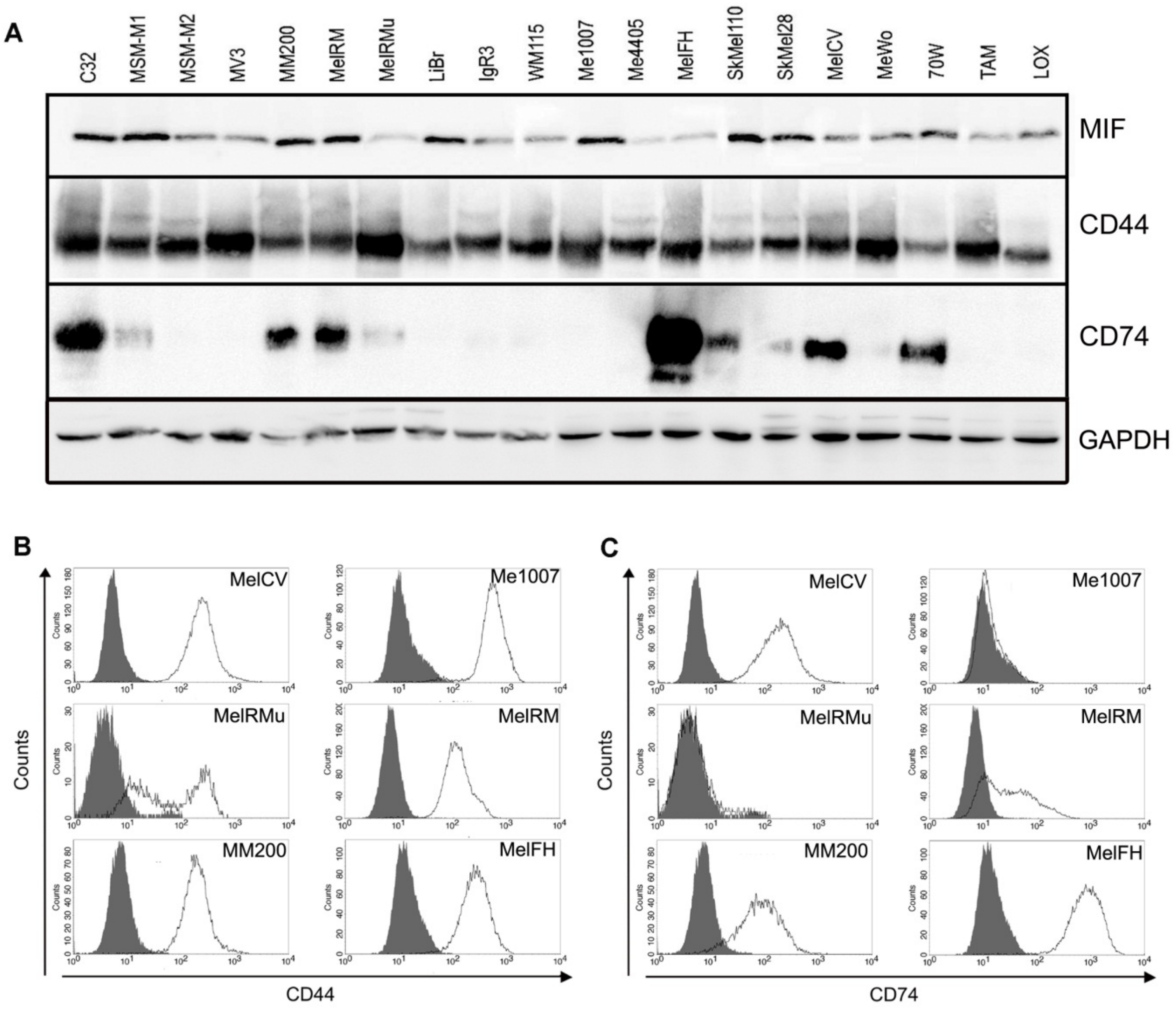

Figure S2. Expression of MIF and its receptors CD44 and CD74 in a human melanoma cell line panel.

(A) Western blots showing immunoreactive bands for MIF (~12.5 kDa detected with R&D #MAB289), CD44 (~85-150 kDa; mAb clone Hermes-3) and CD74 (~34 kDa; mAb clone FMC14) across a panel of 20 human melanoma cell lines. Blotting of samples against GAPDH (~36 kDa) served as a loading control. The cell lines were obtained from the ATCC or isolated from fresh surgical biopsies from patients attending the Sydney and Newcastle Melanoma Units. The MIF receptor, CD74, was detected in 10 out of 20 cell lines, while the co-receptor CD44 was ubiquitously expressed. Receptor expression was also confirmed by cell surface staining and flow cytometric analysis for the six cell lines used throughout this study.

(B and C) Specific antibody staining (open histogram) is shown overlayed over staining using a control antibody (solid histogram). (B) Consistent with the Western blotting results, all the cell lines displayed positivity for CD44. (C) Similarly, CD74 expression levels detected by flow cytometry were consistent with the expression levels observed by Western blotting.

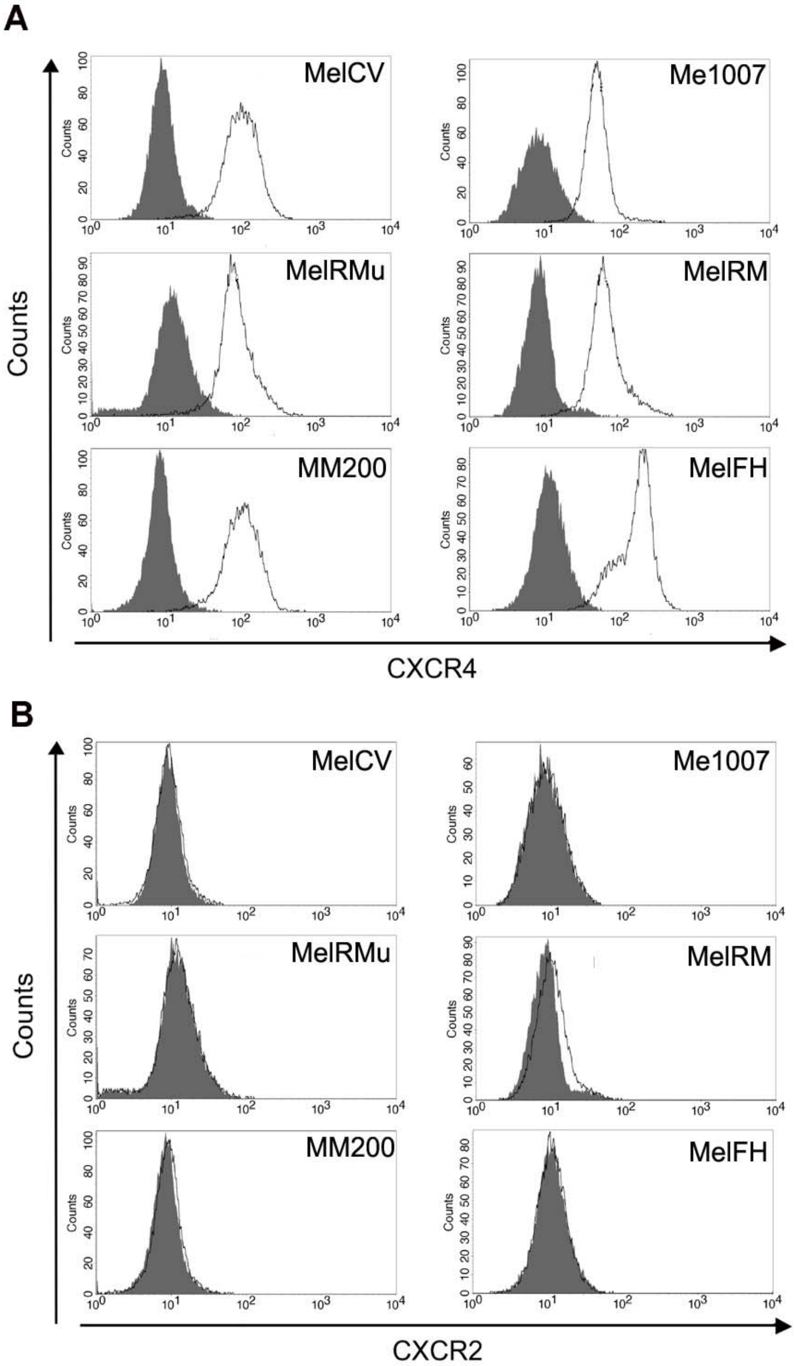

Figure S3. Chemokine receptor expression in human melanoma cell lines.

(A) CXCR4 and (B) CXCR2 expression was assessed by cell surface staining and flow cytometry analysis (using mAb clones Abcam #Ab2074 and BD Pharmingen #555932, respectively). The cells were stained with specific antibodies compared to control antibodies (open histogram versus solid histogram respectively). (A) CXCR4 was present in all the cell lines analysed while (B) CXCR2 expression was absent in all cell lines.
